# We are Legend

**DOI:** 10.3201/eid1409.080682

**Published:** 2008-09

**Authors:** Julian W. Tang

**Keywords:** HIV, NRTIs, NNRTIs, PIs, INIs

We are HIV. Our family is ancient.
Out of Africa,
Monkey to man,
From the trees and forests,
To the towns and cities.
We are here.

For we are HIV, we are legion.
Our children are billions,
Our home, in your defenses,
In your blood, your brain,
Your saliva, your semen.
We are everywhere.

For we are HIV, we are immortal.
We are part of you,
And you of us,
We live with you, but
May not die with you.
We go on.

For we are HIV, we are travelers.
From lover to lover,
Mother to baby,
Donor to blood bank,
Blood bank to patient,
We follow you.

For we are HIV, we evolve.
NRTIs, NNRTIs, PIs, INIs,
New designs, new drugs,
Bring it on, bring it on,
Q151M, K103N, L90M.
We adapt, we survive.

We are HIV. We consume.
Your resources, your time,
Your hope, your lives,
Your new drugs are easy.
Where are your vaccines?
Can you stop us? We will see.

